# Drug-Induced Severe Cutaneous Adverse Reactions: Insights Into Clinical Presentation, Immunopathogenesis, Diagnostic Methods, Treatment, and Pharmacogenomics

**DOI:** 10.3389/fphar.2022.832048

**Published:** 2022-04-20

**Authors:** Therdpong Tempark, Shobana John, Pawinee Rerknimitr, Patompong Satapornpong, Chonlaphat Sukasem

**Affiliations:** ^1^ Division of Dermatology, Department of Pediatrics, Faculty of Medicine, Chulalongkorn University, Bangkok, Thailand; ^2^ The Pediatrics-Thai Severe Cutaneous Adverse Drug Reaction (Ped-Thai-SCAR) Research Group, Bangkok, Thailand; ^3^ Division of Pharmacogenomics and Personalized Medicine, Department of Pathology, Faculty of Medicine Ramathibodi Hospital, Mahidol University, Bangkok, Thailand; ^4^ Laboratory for Pharmacogenomics, Somdech Phra Debaratana Medical Center (SDMC), Ramathibodi Hospital, Bangkok, Thailand; ^5^ The Thai Severe Cutaneous Adverse Drug Reaction (Thai-SCAR) Research Group, Bangkok, Thailand; ^6^ Division of Dermatology, Department of Medicine, Faculty of Medicine, Skin, and Allergy Research Unit, Chulalongkorn University, Bangkok, Thailand; ^7^ Division of General Pharmacy Practice, Department of Pharmaceutical Care, College of Pharmacy, Rangsit University, Pathum Thani, Thailand; ^8^ Excellence Pharmacogenomics and Precision Medicine Centre, College of Pharmacy, Rangsit University, Pathum Thani, Thailand; ^9^ Pharmacogenomics and Precision Medicine, The Preventive Genomics & Family Check-up Services Center, Bumrungrad International Hospital, Bangkok, Thailand; ^10^ MRC Centre for Drug Safety Science, Department of Pharmacology and Therapeutics, Institute of Systems, Molecular and Integrative Biology, University of Liverpool, Liverpool, United Kingdom

**Keywords:** SCARs, pharmacogenomics, PGx implementation, Thailand, immunopathogenesis of SCARs, risk factors

## Abstract

SCARs are rare and life-threatening hypersensitivity reactions. In general, the increased duration of hospital stays and the associated cost burden are common issues, and in the worst-case scenario, they can result in mortality. SCARs are delayed T cell-mediated hypersensitivity reactions. Recovery can take from 2 weeks to many months after dechallenging the culprit drugs. Genetic polymorphism of the *HLA* genes may change the selection and presentation of antigens, allowing toxic drug metabolites to initiate immunological reactions. However, each SCARs has a different onset latency period, clinical features, or morphological pattern. This explains that, other than *HLA* mutations, other immuno-pathogenesis may be involved in drug-induced severe cutaneous reactions. This review will discuss the clinical morphology of various SCARs, various immune pathogenesis models, diagnostic criteria, treatments, the association of various drug-induced reactions and susceptible alleles in different populations, and the successful implementation of pharmacogenomics in Thailand for the prevention of SCARs.

## Introduction

Cutaneous adverse drug reactions (CADRs) include a wide range of clinical symptoms, from moderate and self-limited cutaneous eruptions such as maculopapular exanthema (MPE) to severe cutaneous adverse drug reactions (SCARs) (Alvestad et al., 2007). CADRs are delayed-type hypersensitivity reactions. Usually, the inflammation sign starts within 12–24 h or sometimes after many days of drug exposure (Patel et al., 2014a). Though these reactions are usually mild and can be treated by reducing the dose of the culprit drug or by simple treatments, SCARs need hospitalization. In very severe cases, intensive care is necessary. Other concerns for patients with SCARs include longer hospital stays if they are already hospitalized, extra-economic load, and death (Campos-Fernández et al., 2005).

SCARs can manifest in a variety of ways, the most common SCAR is Stevens−Johnson syndrome/toxic epidermal necrolysis (SJS/TEN), followed by drug reaction with eosinophilia and systemic symptoms (DRESS) and acute generalized exanthematous pustulosis (AGEP). Antibiotics are the most commonly implicated medications of SCARs, followed by anti-epileptic drugs (AEDs), allopurinol, and non-steroidal anti-inflammatory drugs (NSAIDs). Genetic mutations, in addition to clinical and non-clinical factors, have a significant influence on the development of SCARs (Fowler et al., 2019).

Based on our ThaiSCAR research experience, this review not only provides a pragmatic understanding of SCARs but also recommends pharmacogenetic testing (PGx) before treatment. Our goal is to inform about the prevalence of SCARs in different ethnic groups; how clinical features differ for different SCARs; what pathogenesis mechanisms each SCARs may have; diagnostic challenges and established diagnostic criteria; drug-specific, phenotype-specific, and ethnicity-specific pharmacogenetic variants associated with SCARs; and the importance of PGx testing in predicting and preventing SCARs.

## Epidemiology

CADRs affect 1–3% of people in developed nations and 2–5% of people in developing countries (Bigby, 2001; Craig et al., 2001). SCARs are rare reactions, yet they have a significant mortality rate. SCARs have a global frequency of 0.4–1.2 cases per million per year, with a 14–70 percent death rate (Verma et al., 2013). According to a prospective population-based registry in Germany, the annual incidence of SCARs was 1.53–1.89 per million People (Mockenhaupt, 2012a). However, the incidence rate of SCARs is reported to be higher among East Asians. The prevalence of SCARs was reported to be 0.32/1000 hospitalizations in a Beijing study (Li and Ma, 2006). Another prospective study in Singapore found a 5.2% SCAR incidence and a 0.09/1000 hospital death rate due to allergies (Thong et al., 2003). SJS/TEN is the most prevalent SCAR among SCARs, followed by DRESS, AGEP, SJS/TEN accounted for nearly 67% of the 755 SCAR cases documented in the Korean ADRs system, followed by DRESS at 32.7% (Kang et al., 2019).

Many epidemiological studies have found the same type of SCARs occurrence pattern in different populations. These events, however, may vary depending on the drug. According to the same Korean ADRs system study, DRESS was the most prevalent SCARs for allopurinol, with an 11.3% incidence rate compared to SJS/TEN (10.2%) (Kang et al., 2019). In case of antibiotics and phenytoin (PHT), AGEP and DRESS were prevalent SCARs. Among many drug categories, antibiotics are the ones that reported the highest number of SCARs in many studies (Choon and Lai, 2012; Loo et al., 2018). The second most common pharmacological class involved in SCARs is AEDs. Antibiotics (29.5%) and anticonvulsants (24.1%), for example, were the most common causal drug classes in cohort research conducted at Fujian Medical University (Loo et al., 2018). A similar implication pattern on SCARs was discovered in retrospective research conducted in Singapore. Though no gender disparity in the occurrence of SCAR was found in some studies (Su and Aw, 2014), Patients' age ranged from 40.8 to 56 years old (21 years), with a mean age of 40.8 years, are vulnerable to SCARs (Patel et al., 2013; Yang et al., 2018).

### Clinical Manifestation

#### SJS/TEN

In about one-third of cases, SJS/TEN is preceded by non-specific prodromal symptoms lasting for 1–7 days. For the first few days, SJS/TEN is characterized by erythroderma. The erythrodermic rashes then develop into erythematous macules with purpuric centers and blisters in a symmetrical pattern. These severe cutaneous lesions aggressively detach the epidermis and cause mucous membrane erosion. Stinging eyes, photophobia, malaise, fever, headache, anorexia, sore throat, and pseudo-membrane formation of the eyes and genitalia are other pertinent symptoms of SJS/TEN. SJS and TEN can be distinguished by the extent of the epidermal detachment. As a result, the degree of body surface area (BSA) involvement is determined by adding all blisters and skin detachment together (Saha et al., 2012; Tan and Tay, 2012).

**FIGURE 1:**
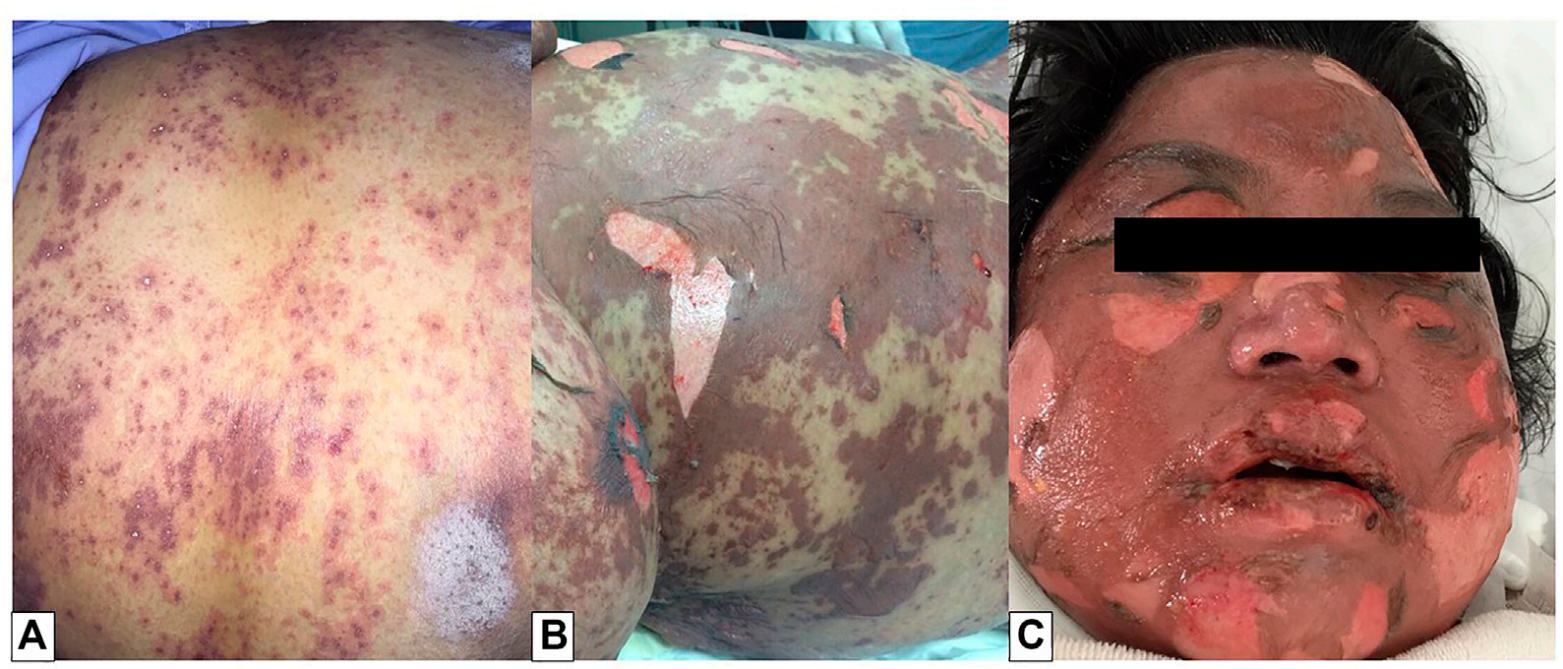
Toxic epidermal necrolysis (TEN). Multiple macules with central dusky red (atypical target lesions) were found on the trunk **(A)**. The lesions progressed to flaccid bullae and large sheets of skin necrosis, leading to diffuse erythema. Nikolsky’s sign was also positive **(B)**. Facial, oral, and ocular involvements were noted **(C)**.

In SJS, SJS/TEN overlapping, and TEN, the BSA of involved skin detachment was less than 10%, 10–30%, and more than 30%, respectively. Nikolsky sign is manifested by a dermal-epidermal cleavage when applying tangential pressure to apparently intact skin, even this sign was not pathognomonic but also helpful to diagnose this condition. At least two mucous membrane involvements were found in 95% of SJS/TEN cases. Edematous, erythematous, and flaccid bullae lesions are other clinical features generally followed by painful, burning sensations in the conjunctivae, oral cavity, and genitalia (Figure 1). The most commonly affected systems in SJS/TEN patients are the respiratory system (pneumonitis, bronchial hypersecretion), gastrointestinal system (transaminitis, hematemesis, gastrointestinal hemorrhage, esophageal stricture), and renal system (micro-albuminuria, hematuria, interstitial nephritis, acute renal failure). In SJS/TEN patients, however, septicemia is the leading cause of death, with *Staphylococcus aureus* and *Pseudomonas aeruginosa* being the most common pathogens. Multiple organ failure is caused by septicemia, hypovolemia, hypoalbuminemia, and transaminitis (Schwartz et al., 2013).

### DRESS

**FIGURE 2:**
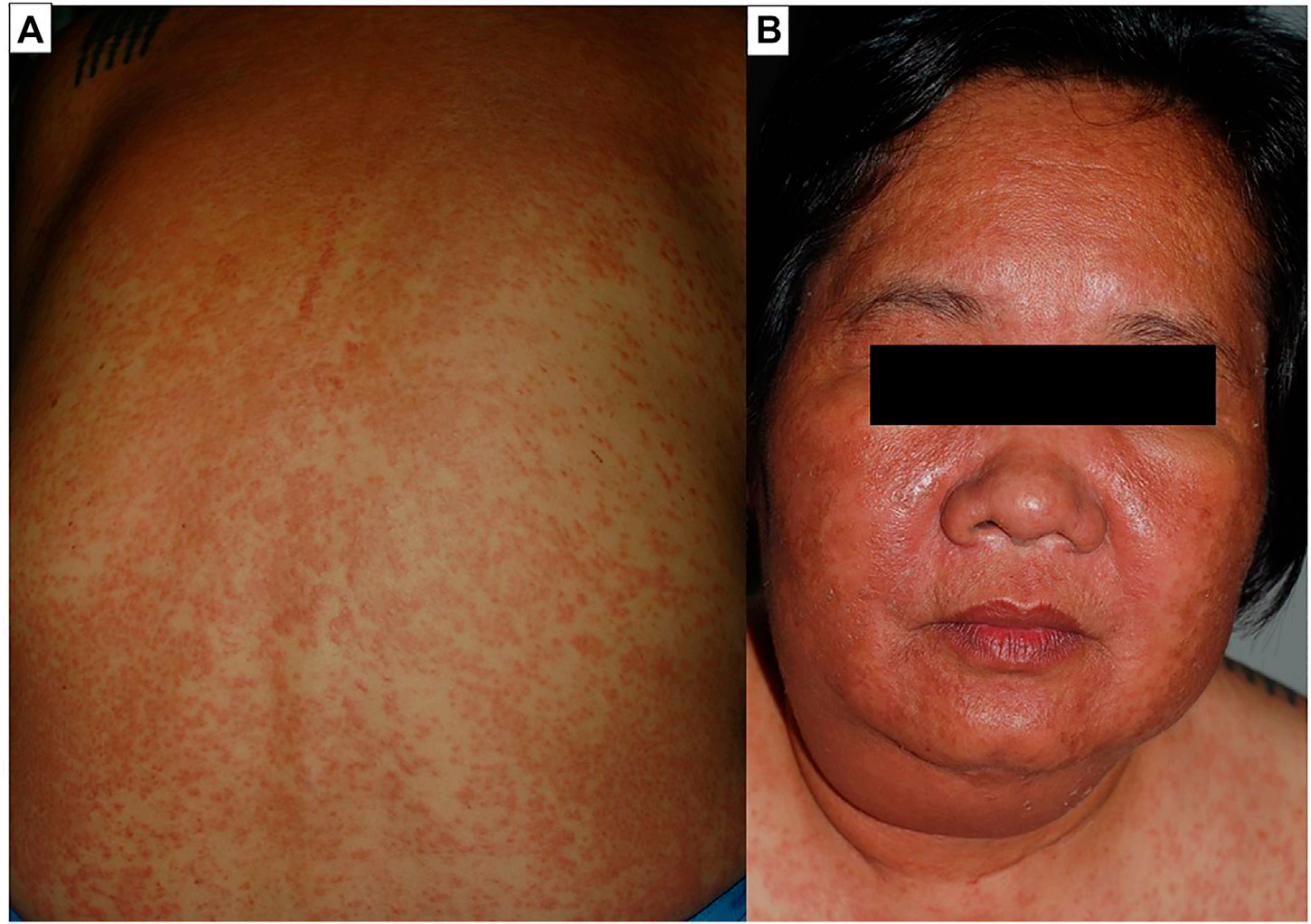
Drug reaction with eosinophilia and systemic symptoms (DRESS). Diffuse maculopapular eruption involving more than 50% of body surface area. The plaques were infiltrated and exhibited follicular accentuation **(A)**. Facial edema is a prominent feature of the syndrome **(B)**.

The most prevalent cutaneous sign of DRESS is a polymorphous maculopapular eruption, which is followed by exfoliation and desquamation. The skin lesion starts on the face, with periorbital and facial edema, and then spreads to the upper torso and lower limbs in 25–76% of cases (Watanabe, 2018). Pustules, purpura, plaques, blisters, target-like lesions, urticarial lesions, and lichenoid lesions are also seen in DRESS patients (Figure 2).

The natural course of dermatologic presentation in DRESS is frequently diverse and recurs for several weeks, with an average duration for clinical improvement of 6–9 weeks (average 21.8 days) (Spriet and Banks, 2015a; Kim et al., 2020a). The recurrence, on the contrary, could have been prompted by exposure to a drug that was unrelated to the offending substances and had weaker symptoms than the earlier manifestation. Mucosal involvement can be found in about 56% of cases at lip and mucosa (Kardaun et al., 2013a). Internal organ involvement, such as lymphadenopathy and hematological disorders (leukocytosis, eosinophilia, atypical lymphocytes, thrombocytopenia), is common in DRESS. The most prevalent systemic manifestation is hepatic involvement, followed by renal, pulmonary, neurological, and cardiac involvement (Cacoub et al., 2011). The most serious sequela is hepatic necrosis, which causes severe liver injury or sudden liver failure, followed by coagulopathy and sepsis, which is the major cause of death (Husain et al., 2013a). Endocrine involvement is uncommon, although it has long-term consequences. Thyroid gland is the most typically impacted gland (Kano et al., 2010; Tempark et al., 2022).

### AGEP

**FIGURE 3:**
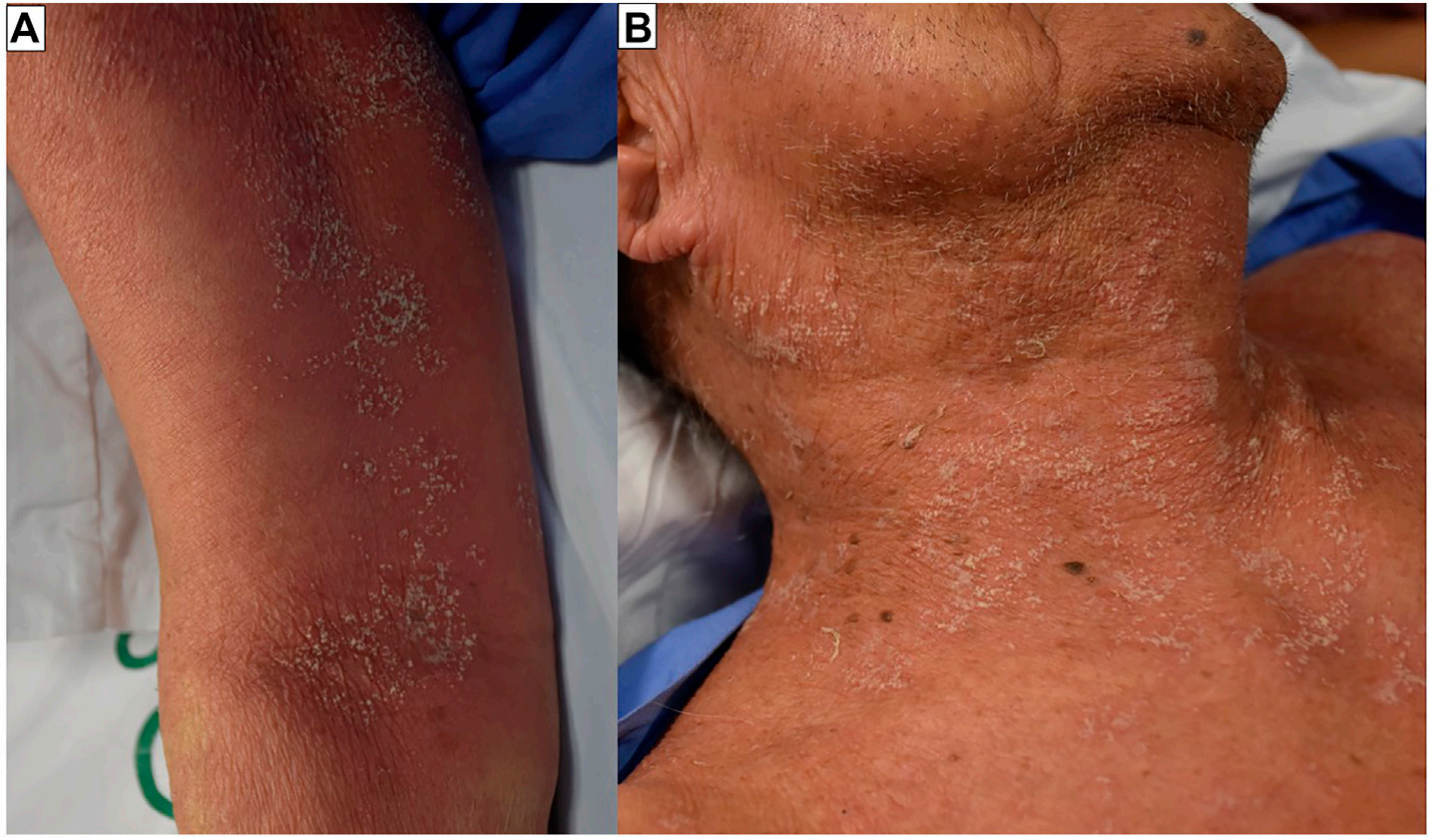
Acute generalized exanthematous pustulosis (AGEP). Multiple pin-head sized, non-follicular pustules on edematous, homogenous, poorly demarcated, erythematous background. The lesions were found mainly on the folds of the body **(A)**. The pustules can become confluent **(B)**.

AGEP manifests as a fever with rapidly spreading multiple pinhead-sized, sterile, non-follicular pustules on an erythematous and edematous base, particularly in the intertriginous zones of the neck, sub-mammary, inguinal, axillary area, trunk, and upper extremities. Pruritus with or without a burning sensation is reported by some patients. TEN-like AGEP (TEN-AGEP overlap) and AGEP-DRESS overlap are two examples of atypical systemic presentation. Atypical localized AGEP, also known as atypical localized exanthematous pustulosis (ALEP), is a kind of AGEP that affects the face, neck, and chest (Villani et al., 2017) (Figure 3). Fever and leukocytosis are the first symptoms, followed by isolated non-follicular pustules at the site. Ryder ENC proposed the Acute localized exanthematous pustulosis diagnosis criteria recently (Ryder and Perkins, 2018). Leukocytosis (>10,000/ml) and an increased neutrophil count (neutrophilia >7,000/ml) are found in the majority of AGEP patients. Eosinophilia is prevalent in about 30% of patients. Hepatic dysfunction, renal insufficiency, respiratory distress, agranulocytosis, and lymphadenopathy are examples of multi-organ involvement that have been documented in roughly 17% of patients (Szatkowski and Schwartz, 2015a).

**FIGURE 4:**
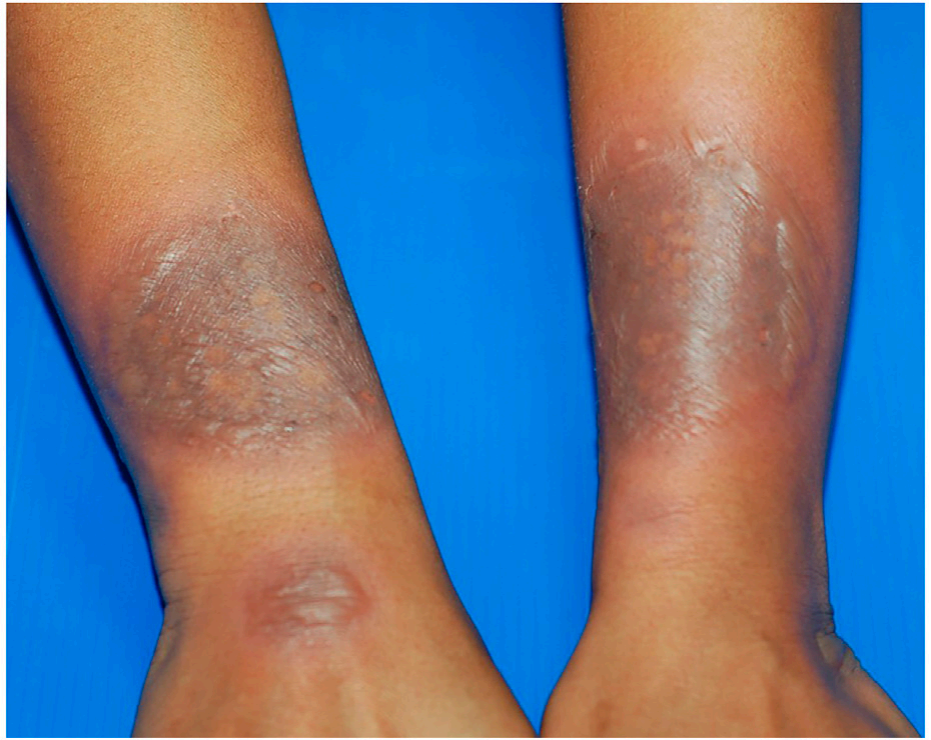
Generalized bullous fixed drug eruption (GBFDE). Multiple large and well-demarcated round to oval plaques with blisters formation. The lesions were found at different sites of the body. Fewer than two sites of the mucous membrane were involved. The patient has experienced a similar reaction in the past.

GBFDE is differentiated from FDE by the occurrence of widespread bullae and erosions. The most commonly affected locations are the head, neck, anterior and posterior trunck, upper and lower limbs, and genitalia. The 10% skin detachment is discovered in several cases (Figure 4). The onset of cutaneous symptoms can help distinguish GBFDE from SJS/TEN. FDE symptoms, for example, usually appear within 30 min to 24 h after exposure to the culprit drug, whereas SJS/TEN symptoms appear 1 to 3 weeks. Another noteworthy distinction is the affliction site; because FDE recurs at the same location, more FDE/GBFDE patients have a positive past allergy history compared to SJS/TEN patients (Mitre et al., 2017).

## Investigation

Skin patch testing, lymphocyte transformation test (LTT), and ELISpot assay were three effective ways for finding the culprit drug for SCARs (Husain et al., 2013b). Positive patch tests produce a clinically red spots on the skin due to the recruitment of inflammatory cells and can be used to prove the activation of drug-specific T cells. This test should be carried out on the previously afflicted site. However, the type of drug, drug concentration, formulation (intravenous powder, capsule), diluents used, patch testing site, and duration after exposure are different variables that can affect the interpretation of patch test results. According to the recommendation of the International Contact Dermatitis Research Group (ICDRG), a patch test should be performed during the 6 months following DRESS and at least 1 month after cessation of immunosuppressant (Barbaud, 2014).

Multiple factors could affect the sensitivity and specificity of patch testing. A positive patch test is useful to confirm an inflammatory cutaneous hypersensitivity reaction, but a negative test cannot exclude the causative drugs (Husain et al., 2013b). Studies showed a higher positive predictive value for the patch test than the negative predictive value. For example, among AEDs, the patch test of CBZ shows a high positive predictive value of about 80%–90% for DRESS (Shanbhag et al., 2020), but it ranges only 10%–20% for phenobarbitol (Elzagallaai et al., 2009; Ohtoshi et al., 2014; Correa-de-Castro et al., 2016a). According to a multicenter study conducted by Barbaud et al., the comparative value of patch testing for the diagnosis of AGEP, DRESS, and SJS/TEN was 58%, 64%, and 24%, respectively (Barbaud, 2014).

The lymphocyte transformation test (LTT) is an *in vitro* test for delayed hypersensitivity reaction that measures radioactive thymidine incorporation into the DNA of proliferating cells to detect regulatory T cell activation after pharmacological stimulation. In DRESS and drug-induced hypersensitivity syndrome (DIHS), these tests can be conducted from 5–7 weeks to 1 year after the commencement of the rash (Kano et al., 2007; Fernando, 2014). However, for SJS/TEN, LTT must be performed within the first week of rash development (Kano et al., 2007; Ferrandiz-Pulido and Garcia-Patos, 2013). Although drug identification was not done on a regular basis, the LTT sensitivity was positive in 21%–56% of these patients (Kano et al., 2007; Porebski et al., 2013), and it is much higher for AGEP, DRESS (Hassoun-Kheir et al., 2016; Pinho et al., 2017). ELISpot assay is the *in vitro* test to access functional Tregs on cytokine production in the acute phase of DRESS by drug-specific interferon-γ releasing cell. In cultured peripheral blood mononuclear cells (PBMCs) with a suspected drug, the IFN-ELISpot assay evaluates IFN produced by DRESS cells. This assay is drug-specific; several studies, for example, beta-lactam-induced maculopapular rash (Suthumchai et al., 2018a) and allopurinol-induced SCARs (Rozieres et al., 2009), have verified the drug-specific release of IFN (Klaewsongkram et al., 2016).

## Causative Agents and Risk Factors of SCARs

The most common cause of SJS/TEN is drug administration; however, other reasons may account for 5%–15% (Sassolas et al., 2010a). The prevalence of SCARs varies according to the medications used and the age of the patient. Common culprit drugs in DRESS include AEDs (carbamazepine, lamotrigine, phenobarbital, phenytoin), antibiotics (amoxicillin, ampicillin, azithromycin, levofloxacin, minocycline), anti-tuberculosis agents (ethambutol, isoniazid, rifampicin, pyrazinamide), analgesics (diclofenac, ibuprofen, aspirin, celecoxib), sulfonamides (dapsone, sulfamethoxazole-trimethoprim, sulfasalazine), anti-viral (abacavir, nevirapine), anti-hypertensive (amlodipine, captopril), allopurinol, and amitriptyline. Aminopenicillin, quinolones, hydroxychloroquine, sulfonamides, terbinafine, and diltiazem were the most common causes of AGEP in the EuroSCAR study. On the contrary, corticosteroids, macrolides, oxicam, NSAIDs, and antiepileptic medications have a weak connection with AGEP (Szatkowski and Schwartz, 2015b).

The onset of SCARs varies depending on the type of SCARs. For example, the onset of DRESS patients ranges from 2 to 8 weeks (average 22.2 days, range 0.42–53 days) (Bocquet et al., 1996; Schwartz et al., 2013; Kim et al., 2020b), whereas it ranges from 24 to 48 h (1 hour to 25 days) (Halevy, 2009; Hadavand et al., 2020). Similarly, there were disparities between children and adults in terms of the causative substance, associated causes, and severity. Sulfonamides, aromatic anticonvulsants (carbamazepine, phenytoin, and phenobarbital), penicillin, and NSAIDs were prevalent implicated drugs in children, while allopurinol, oxicam, NSAIDs, and nevirapine induced SCARs were common in adults (Ferrandiz-Pulido and Garcia-Patos, 2013). After being exposed to the probable drugs, the rash usually appears within 8 weeks (Ferrandiz-Pulido and Garcia-Patos, 2013). In the prior literature, carbamazepine has been identified as the most common causal agent in adult and pediatric DRESS patients (Bocquet et al., 1996; Cacoub et al., 2011; Spriet and Banks, 2015b). However, according to Oberlin KE et al., antibiotics (cephalosporin, trimethoprim-sulfamethoxazole, and vancomycin) were the most common cause of pediatric DRESS (Zhou et al., 20161936; Oberlin et al., 2019).

Identifying the risk factors and understanding their pathogenesis can help optimize therapy. The risk factors are generally 1) immune-mediated: HLA allele genetic polymorphisms can cause these reactions; these variants can differ in every population, drug, and phenotype; and they are metabolism independent and 2) non-immune mediated: these reactions can be caused by genetic abnormalities in genes that encode enzymes involved in drug metabolism, as well as patient, disease, and drug-related variables (Galindo et al., 2002).

### Genetic Factors

Roujeau et al. were the first to report an association between HLA and SJS in 1986, the year after he confirmed an association between HLA and TEN. The suspected alleles of SCARs could be drug-specific, ethnicity-specific, and phenotypic-specific. Abacavir and HLA-B* 57:01 were the subjects of the first groundbreaking studies on immune-mediated reactions (Mallal et al., 2002; Hughes et al., 2009). Many clinical guideline agencies recommended the pre-prescription genotyping of HLA-B* 57:01 before initiating the abacavir treatment. The second breakthrough research in ADR-pharmacogenetics was reported by Chung et al., showing a strong association between SJS/TEN and HLA-B* 15:02 among the Han Chinese population in Taiwan in 2004 (Chung et al., 2004). Later, this association was confirmed in many other populations, including Chinese (Wang et al., 2011), Thai (Tassaneeyakul et al., 2010a), Malaysians (Chang et al., 2011a), Indian (Mehta et al., 2009), Vietnamese (Nguyen et al., 2015a), and Indonesians (Yuliwulandari et al., 2017). On the contrary, the involvement of HLA-A* 31:01 in CBZ induced SJS/TEN has been demonstrated in Western and Japanese populations (Kashiwagi et al., 2008a; McCormack et al., 2011). As a result, the FDA recommended a pharmacogenetic (PGx) biomarker classification system based on dependability, scientific evidence, and clinical utility (US Department of Health and Human Services, United States Food and Drug Administration, August 2011) (US Department of Health and Human Services, 2011). It provides a valuable framework for evaluating putative biomarkers, although the marker has less positive predictive value. Table 1 shows the most associated *HLA* alleles with SCARs in various population.

**TABLE 1:** Genetic factors (HLA) associated with various SCARs in different populations.

Drug	Ethnic group	Type of SCARs	HLA variants	References
Abacavir	Caucasian	AHS	HLA-B*57:01	Sousa-Pinto et al. (2015)
	Australian	AHS	HLA-B*57:01	Sousa-Pinto et al. (2015)
	Hispanic	AHS	HLA-B*57:01	Cristallo et al. (2011)
	Asians	AHS	HLA-B*57:01	Gonçalo et al. (2013)
Allopurinol	Caucasian	SCARs	HLA-B*58:01 39.11(4.49–340.51) 88	Hung et al. (2005)
			HLA-C*03:02	
			DRB1*15:02	
			DRB1*13:02	
	Han Chinese	SCARs	HLA-B*33:03	Niihara et al. (2013)
			HLA-B*58:01 39.11(4.49–340.51) 88	
			HLA-C*03:02	
	Japanese	SCAR/EM	HLA-B*58:01	Kang et al. (2011)
	Korean	SCAR	HLA-B*33:03	Tassaneeyakul et al. (2009a)
			HLA-B*58:01 39.11(4.49–340.51) 88	
			HLA-C*03:02	
	Thai	SCAR	HLA-B*58:01	Kim et al. (2013)
Anti-TB	Korean	DRESS	HLA-C*04:01	Kim et al. (2013)
CBZ	Caucasian	SJS/TEN	HLA-A*31:01	Genin et al. (2014)
				Hung et al. (2006)
		DRESS	HLA-A*31:01	Hung et al. (2010a)
			HLA-B*07:02	
	Han Chinese	SJS/TEN	HLA-A*24:02	He et al. (2013)
			HLA-A*33:03	Tangamornsuksan et al. (2013a)
			HLA-B*15:02	Shi et al. (2012)
			HLA-B*15:11	Genin et al. (2014)
		DRESS	HLA-A*31:01	Shi et al. (2012)
			HLA-B*51:01	
	India	SJS/TEN	HLA-B*15:02	Khor et al. (2014)
	Japanese	SJS/TEN	HLA-A*02:06	Kaniwa et al. (2010)
				Kashiwagi et al. (2008)
			HLA-B*15:11	Hsiao et al. (2014)
	Korean	SCARs	HLA-A*31:01	Kim et al. (2011)
		SJS/TEN	HLA-B*15:11	
	Malay	SJS/TEN	HLA-B*15:02	Chang et al. (2011b)
	Thai	SJS/TEN	HLA-B*15:02	Zhang et al. (2013)
	Vietnamese	SJS/TEN	HLA-B*15:02	Tangamornsuksan et al. (2013)
		SJS/TEN	HLA-B*46:01	
Cold medications	Indian	SJS/TEN	HLA-B*44:03	Locharernkul et al. (2008)
	Brazilian		HLA-B*44:03	Nguyen et al. (2015b)
	Korean		HLA-A*02:06	
Co-trimoxazole	Thai	SJS/TEN	HLA-B*15:02	Ueta et al. (2014a)
			HLA-C*06:02	
			HLA-C*08:01	
Dapsone	Chinese	HSS	HLA-B*13:01	Ueta et al. (2014b)
	Southern Chinese	DIHR	HLA-B*13:01	Ueta et al. (2014b)
	Han Chinese	DRESS	HLA-B*13:01	Ueta et al. (2014b)
			HLA-C*03:04	Kongpan et al. (2015)
	Thai		HLA-B*13:01	Tempark et al. (2017)
Lamotrigine	Caucasian	SCAR	HLA-A*68:01	Zhang et al. (2013)
			HLA-B*58:01	
			HLA-DQB1*06:09	
	Han Chinese	SJS/TEN	HLA-B*13:01	Park et al. (2020)
			HLA-B*15:02	
			HLA-C*08:01	Kazeem et al. (2009)
			HLA-DRB1*16:02	
Methazolamide	Han Chinese	SJS/TEN	HLA-B*59:01	Yang et al. (2016)
			HLA-C*01:02	
	Korean	SJS/TEN	HLA-B*59:01	Kim et al. (2010)
			HLA-C*01:02	
Nevirapine	Black	SJS/TEN	HLA-C*04:01	Carr et al. (2013)
	Brazilian	SJS/TEN	HLA-B*44:03	
	Indian	SJS/TEN	HLA-B*44:03	
	Korean	SJS/TEN	HLA-A*02:06	
	Malawian	SJS/TEN	HLA-C*04:01	Carr et al. (2013)
Phenytoin	Han Chinese	SJS/TEN	HLA-B*13:01	Hung et al. (2010b)
			HLA-B*15:02	
			HLA-C*08:01	
			HLA-DRB1*16:02	
	Thai	SJS/TEN	HLA-B*15:02	
Sulfamethoxazole	Thai	SJS/TEN	HLA-B*15:02	Kongpan et al. (2015)
			HLA-C*06:02	
			HLA-C*08:01	
Sulfasalazine	Han Chinese	DRESS	HLA-B*07:02	Yang et al. (2014)
			HLA-B*13:01	
			HLA-B*15:05	
			HLA-B*39:01	
			HLA-B*58:01	
Zonisamide	Japanese	SJS/TEN	HLA-A*02:07	Kaniwa et al. (2013)

AHS, abacavir induced hypersensitivity; DIHR, drug-induced hypersensitivity reactions; DRESS, drug reaction with eosinophilia and systemic symptoms; EM, erythema multiforme; SCARs, severe cutaneous adverse reactions; SJS, Steven–Johnson syndrome; TEN, toxic epidermal necrolysi; HSS, hypersensitivity syndrome.

### Non-Genetic Factors

Identifying the risk alleles and their predictive positive and negative values for the occurrence of SCARs can be helpful in the optimization of therapy. However, defects in several gene loci cannot be the only reason for any SCARs. Recognizing the other non-genetic factors and the level of their association with each SCARs can help increase specificity (Table 2). Overall, to establish a highly specific treatment strategy, both genetic and non-genetic factors should be studied in depth. For example, the incidence of SJS among children on lamotrigine (LTG) treatment has been estimated to be as high as 1:100 compared to adults 1:1000 (Dreifuss et al., 1987a). Recent Thai research, on the contrary, has found no link between age and the occurrence of AED-induced CADRs (Yampayon et al., 2017a; Sukasem et al., 2020a). However, in gender, women are at high risk of developing CADRs (Tran et al., 1998).

**TABLE 2:** Non-genetic factors associated with SCARs.

Parameter	Drugs/factors	Effect	References
Age	AEDs	SJS common among pediatrics	Raucci et al. (2013)
	Antibiotics		
	CBZ	Twenty-five percent of drug withdrawal happened among new-onset elderly epileptic patients	Brodie et al. (1999)
	AEDs	Elderly patients experience skin reactions from AEDs at higher rates	Sullivan and Shear (2002)
	LTG	The incidence of SJS in children on LTG treatment has been estimated as high as 1:100 compared to adults 1:1,000	Dreifuss et al. (1987b)
Gender	Females	SJS reported at higher rates among females than males. Striking difference was seen among males (8%) and females (19%) with *p* < 0.001	Schmitz and Bergmann (2007)
Family history (FHx)	Positive	The history of drug allergy and family history were shown to be associated with CADR	Asakura et al. (2001)
			Campos-Fernández et al. (2005)
Ethnicity	PHT	SJS/TEN, the incidence rate usually was higher among East Asians, especially Han Chinese	Yampayon et al. (2017)
Obesity	Obese	Patients with >30 BMI reported high numbers of CADRs (50%)	Campos-Fernández et al. (2005)
Radiation therapy		Increased risk of PHT-induced SJS/TEN when PHT treatment and whole-brain radiotherapy are combined	Al-Quteimat (2016)
Concurrent drug use	PHT	VPA increases the risk of PHT-induced rashes	Alvestad et al. (2007)
			Cloyd (1991)
			Maggs et al. (2000)
			Anderson et al. (2015)
	PHT	Omeprazole was found to be more frequent in PHT-induced DRESS/DHS	Yampayon et al. (2017)
Multiple anti-epileptics	AEDs	More than three AEDs increase the risk of CADR, especially with the aromatic AEDs	Joshi et al. (2017)
	PHT		John et al. (2021a)
Underlying diseases		Patients with malignancy DM, HIV, SLE HTN were at risk of SJS/TEN	Patel et al. (2014)
Starting dose	AEDs	The starting dose and incidence of CADRs are particularly evident for LTG, CBZ, and PHT, especially the higher starting dose	Messenheimer et al. (1998)

AEDs, antiepileptic drugs; BMI, body mass index; CADRs, cutaneous adverse drug reactions; DM, diabetes mellitus; HIV, human immunodeficiency virus; HTN, hypertension; LTG, lamotrigine; PHT, phenytoin; SJS, Stevens–Johnson syndrome; SLE, systemic lupus erythematosus; TEN, toxic epidermal necrolysis; VPA, valproic acid.

A study conducted by Alvestad et al. reported that a striking difference was seen among males (8%) and females (19%) with *p* < 0.001 (Alvestad et al., 2007). The explained reasons for this are that the immunological process of rash is influenced by sex hormones. The female sex steroids augment the immune response both pathologically and physiologically, whereas androgens inhibit the inflammatory response more than endogenous glucocorticoid (Talal, 1987; Da Silva, 1999; Osman, 2003).

The history of drug allergy and family history were shown to be associated with SCARs. A study conducted by Asakura et al. (2001) for 5 years reported that 14% of patients had a positive family history for CADRs and Campos-Fernández et al. (2005) reported 23% of family history. The risks for CADRs vary as per the patients’ ethnicity. The incidence rate was usually higher among East Asians, especially Han Chinese. A study from Thailand confirmed that Chinese ethnicity was associated with phenytoin- (PHT-) induced SJS/TEN (Yampayon et al., 2017a). The drug interactions between concurrent drugs may cause CADRs. Concurrent administration of valproic acid (VPA) and LTG increases the risk for CADRs. The reason for it is the inhibiting effect of VPA on uridine diphosphate glucuronyltransferase. According to a 2007 study by Alvestad et al. (2007b), administering VPA with PHT enhances the likelihood of PHT-induced rashes by displacing PHT from protein binding sites and perhaps accumulating reactive PHT metabolites.

Patients comorbid can be a predisposition to CADRs. A systematic review study from India has reported that the commonly reported comorbid conditions were diabetes mellitus (DM) (0.84%), human immunodeficiency virus (HIV) (0.65%), systemic lupus erythematosus (SLE) (0.56%), and hypertension (HTN) (0.28%) in CADRs patients (Patel et al., 2014b).

In the overlap and initiation of SCARs, the underlying illnesses and infection history play a significant role. Infection with *Mycoplasma pneumoniae,* for example, is the second most prevalent cause of SJS in children. However, an infection-induced rash may be less severe than drug-induced SJS/TEN. Coxsackievirus, influenza, herpes simplex virus, cytomegalovirus, parvovirus, varicella-zoster virus, Epstein–Barr virus, measles virus, human herpesvirus types 6 and 7 (HHV-6, HHV-7), *Streptococcus* group A, *mycobacterium*, and *rickettsia* are among the infection-induced SJS/TEN (Kunimi et al., 2011; Ferrandiz-Pulido and Garcia-Patos, 2013), occurring 1 week before the rash appears. Human herpesvirus- (HHV-) 6, HHV-7, Epstein–Barr virus (EBV), and cytomegalovirus are also thought to be responsible for reactivating the DRESS symptoms. Patients should have their viral load and antibody titers checked, especially if they have severe cases that have not responded to systemic corticosteroids. A spider bite, viral infection (Coxsackie B4, cytomegalovirus, parvovirus B19), bacterial infection (*Mycoplasma pneumoniae*, *Chlamydial pneumoniae*, *Escherichia coli*), and parasitic infestation have all been documented as AGEP trigger factors (Szatkowski and Schwartz, 2015c; Hadavand et al., 2020).

## Diagnosis

SCARs have a wide range of clinical characteristics, making diagnosis difficult. Differential diagnoses for SCARs include viral infections, malignancy, hypereosinophilic syndrome, and lymphadenopathy (Fiszenson-Albala et al., 2003). SJS/TEN patients are more likely to have skin detachment and mucosal involvement, whereas DRESS patients are more likely to have organ involvement. Although most reactions are delayed hypersensitivity reactions, the onset latency duration is crucial in SCARs diagnosis. Due to pharmacokinetic features, it may differ depending on the drug or class of drugs. The latency period for phenytoin, for example, might last up to 30 days or more, whereas the latency period of SCARs for CBZ is 7–15 days (John et al., 2021b). When suspecting a substance for SCARs, time and frequency of drug exposure are important factors to examine. AEDs, antibiotics, and allopurinol, for example, are all suspected of being involved with SCARs (Pichler, 2007). As a result, new users of these medications are at risk. As a result, the history and duration of drug exposure, as well as a physical examination, are suspected of diagnosing this disorder.

Because the clinical aspects and duration of each SCARs differ, dermatologists can identify it using a range of established diagnostic criteria. The ALDEN (algorithm of drug causality for TEN) algorithm determines the diagnostic possibility (very likely, probable, possible, unlikely, and extremely unlikely) using six criteria (Sassolas et al., 2010b). As validation criteria for DRESS, the diagnostic criteria, RegiSCAR, Bocquet’s criteria, and J-SCAR were presented (Palmieri et al., 2002). However, there were some differences between these criteria, such as the drug exposure length, evidence of HHV reactivation, and other organ involvement. Clinical signs, histology, and other tests can all be used to diagnose AGEP. Using morphology, clinical history, and histology, the EuroSCAR study group offered four categories of AGEP validation score: no AGEP (score 0), possible (scores 1–4), probable (scores 5–7), and confirmed AGEP (scores 8–12) (Sukasem et al., 2018).

## Treatment

In order to reduce morbidity and death in all SCARs, early diagnosis and drug withdrawal of the potentially causative substance are required. The treatment of SJS/TEN necessitates a multidisciplinary approach. The death rate may drop from 51.4 % to 29.8% when patients are referred to a specialized intensive care unit (ICU) or comparable high-dependency unit and/or the burn unit for optimal supportive care within days (Zimmermann et al., 2017). Supportive treatment typically includes skin mucosa and wound management, air-fluidized beds, thermoregulation, airway protection, fluid replacement, and fluid balance assessment. The need for nutritional assistance, eye care, and pain control cannot be overstated. Secondary bacterial skin infection and septic shock should be monitored to decrease mortality.

Multiple treatments are given, including systemic corticosteroid, intravenous immunoglobulin (IVIG), cyclosporine, anti-tumor necrosis factor (TNF), and cyclophosphamide. There was no standard consensus in terms of the type of immunosuppressive agents (systemic corticosteroids, IVIG, and cyclosporine), efficacy, and dosage of these medications (Huang et al., 2012). Glucocorticoid could probably be considered in some cases. Regardless of the risk of infection or sepsis, a systematic review and meta-analysis revealed that glucocorticoid and cyclosporine seem to be the most promising systemic immuno-modulation therapy that decreases the length of stay in the hospital and increases re-epithelization (Zhang et al., 2020). IVIG lowers the mortality rate caused by SJS/TEN in children (Zhang et al., 2020) compared to adults (Chiou et al., 2008). TNF-inhibitors have also been discovered to be a safe and effective treatment for SJS/TEN. However, the evidence is limited (Teyton et al., 1990a).

Systemic corticosteroid therapy is advised in the early stages of the DRESS diagnosis. Prednisolone (1 mg/kg/day) or an equivalent is frequently given to the patients (Funck-Brentano et al., 2015). Because abrupt discontinuation commonly induces DRESS relapse and prolongs the course of this illness, tapering oral prednisolone should be done gradually for 6–8 weeks to 3 months (Huang et al., 2012) (mean length therapy 56.2 days, range 2–185 days) (Hadavand et al., 2020). Fluid replenishment, an antipyretic drug for fever, correction of electrolyte imbalance, skincare with a proper dressing, and prevention of secondary bacterial infection are the mainstays of treatment. Topical corticosteroids, systemic anti-histamines, and emollients for rashes have been shown to reduce relapse and hospitalization in mild cases of DRESS (Schwartz et al., 2013). The vast majority of AGEP cases just required supportive care. For inflammatory or pruritic lesions, topical corticosteroids might be administered to shorten the clinical course. However, because several organs are affected in severe cases, step-wise treatment with systemic corticosteroids (intravenous/oral) or cyclosporine, as well as adjuvant therapy such as infliximab and IVIG, is employed (Olteanu et al., 2018).

## Prognosis

The prognosis of SJS/TEN patients can be determined using a score of toxic epidermal necrolysis (SCORTEN), which consists of seven independent factors: age, body surface area of skin detachment, underlying malignancy, tachycardia, blood urea nitrogen, serum glucose, and serum bicarbonate. SCORTEN assessments should be done on days 1 and 3 after admission. To assess pediatric SJS/TEN and estimate a mortality rate, pediatric SCORTEN was proposed (Alvestad et al., 2007) (Sassolas et al., 2010c). A study shows that the mortality rates of SJS, SJS/TEN overlap, and TEN were 1%–5%, 5%–25%, and 25%–30%, respectively (Sassolas et al., 2010c).

The most common chronic sequelae were dermatologic and ocular involvements, which were recorded in 88% of SJS patients (Halevy et al., 2008). Atrophic or hypertrophic scars, dyspigmentation, milia, eruptive melanocytic nevi, hyperhidrosis, and xerosis are some of the other dermatological complications. Hair, nail involvement, and mucosal adhesion had also been represented. Chronic inflammation, sicca syndrome, entropion, sub-conjunctival fibrosis, trichiasis, symblepharon, corneal ulceration, permanent dry eye, photophobia, vision loss or impairment, and blindness are common ocular sequelae resulting from changes in the conjunctival epithelium (Kamada et al., 2006; Spriet and Banks, 2015c). The long-term psychosocial effects of SJS/TEN on survivors and their families should be investigated. As long-term sequelae, autoimmune illnesses such as autoimmune thyroiditis, systemic lupus erythematosus, and the Sjögren syndrome might be detected (Kardaun et al., 2013b). The DRESS syndrome is a medication reaction that can be fatal.

DRESS has a better prognosis and lower mortality in children than in adults (Correa-de-Castro et al., 2016b). The death rate in pediatric and adult patients was around 3% and 10%, respectively (Correa-de-Castro et al., 2016b). The mortality rate in the prospective RegiSCAR study was 1.7 percent (Manuyakorn et al., 2016a). However, extensive systemic involvement can considerably increase morbidity and mortality; fulminant hepatitis, coagulopathy, sepsis, and respiratory distress syndrome were the most prevalent causes of DRESS-induced death (Mockenhaupt, 2012b). According to Chen et al., high absolute eosinophil count (>600/UL), thrombocytopenia, pancytopenia, history of chronic renal insufficiency, multiple organ involvement, and underlying illnesses are all bad prognostic markers for this disorder (Fowler et al., 2019). Autoimmune sequelae including thyroid dysfunction, diabetes mellitus, and autoimmune hemolytic anemia should be monitored for several weeks to years after the onset of this condition (Tempark et al., 2022).

The prognosis for AGEP is usually favorable. According to the Euro SCAR study, AGEP has a mortality rate of around 4% (Teyton et al., 1990b). The atypical and severe presentation had a poor clinical prognosis and necessitated systemic treatment.

## Pathogenesis

CADRs can be immediate (mediated by IgE or histamine) or delayed (mediated by histamine) (mediated by T cells). IV drug delivery has been linked to immediate responses such as urticarial rash and angioedema. Because these reactions occur in order with the causative drugs, the diagnosis is rarely suspected. Desensitization should be avoided in this situation due to the high risk-to-benefit ratio. In contrast, the delayed T-cell-mediated effects take anything from 12 h to a week after the first therapeutic dose to manifest. As a result, clinical assessment is critical. SJS/TEN is a serious multi-organ systemic disease with symptoms ranging from a mild skin rash to severe systemic multi-organ disease (Bodmer et al., 1994).

### MHC, TCR, CD4^+^, CD8^+^, and SCARs

Every individual and tissue has its unique MHC. All nucleated cells have MHC-I, whereas APCs such as dendritic cells, macrophages, and neutrophils have MHC-II. CD4^+^ T cells are known as helper T cells, and these exclusively engage with MHC-II, whereas CD8^+^ T cells interact with MHC-I and are known as cytotoxic T cells. MHC I has a full-length alpha chain and a partial beta chain with a presenting site in the middle, whereas MHC-II has both full-length alpha and beta chains with a presenting site in the middle. For successful cell surface antigen presentation, a series of highly coordinated intracellular activities must occur first. The monomorphic protein invariant chain is essential for antigen presentation by MHC class II molecules. From the endoplasmic reticulum, this molecule delivers freshly synthesized class II heterodimers to the endosomal system. As soon as it encounters an acidic environment, this complex will be degraded and dissociate proteolytically, leaving the class II binding site accessible to antigenic peptides produced by external proteins. CLIP (class II-associated invariant chain peptides) requires the assistance of another MHC-encoded protein in order to physically escape the class II binding groove (Neefjes and Ploegh, 1992; Wucherpfennig et al., 2010; Mariuzza et al., 2020).

**FIGURE 5:**
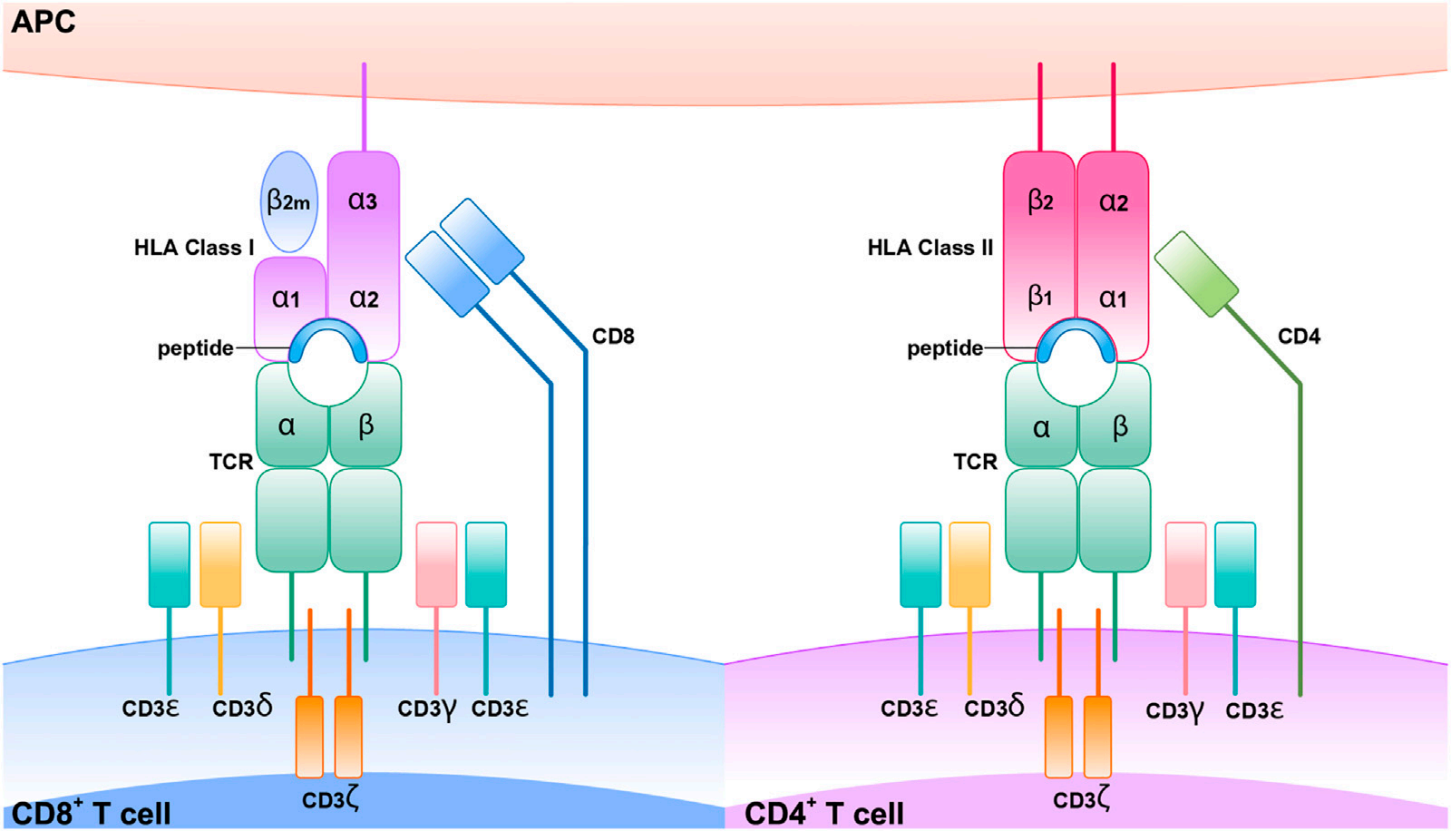
Structure of HLA classes I, II and T cell receptors (TCR).

Cytotoxic T cells and helper T cells are the most influential cells in the immune system. Cytotoxic T cells contain CD8^+^ glycoprotein, releasing perforin that makes pore on the surface of the cell membrane, and granzyme kills the infected cells in a programmed way, called apoptosis. Upon engagement of the MHC-TCR complex, T cell clonal proliferation and differentiation of memory and effector T cells are formed. Among memory T cells, a set of cells are called effectors memory T cells (T_EM_) which are expressed with CD4RO and involved in the surveillance of pathogen re-exposure. Because the activation of these cells requires few co-stimulatory signals, they produce swift immune response upon the reentry of pathogen-specific antigen by producing pro-inflammatory cytokines such as TNF alpha, IL-2, and INF gamma and cytotoxic peptides. T cell response is initiated when the TCR and 1 homodimer z and CD3 (1–3) are collectively expressed as a unit on the surface of the T cell (Chung et al., 2008). For example, CDR1 and CDR2 contact with an alpha unit of MHC, the CDR3 contacts with peptides, showing the greatest sequences variability in the TCR gene. Therefore, the polymorphism of the genes that encode these proteins leads to increased expression of diverse MHC protein, and this may increase the diversity of peptides produced T cells, so diverse pathogens will be recognized and elicit an immune response (Su et al., 2017) (Figure 5).

Granulysin is produced and secreted by both CD8^+^ and natural killer cells (NK cells), which are the key mediators of apoptosis in SJS/TEN. Chung et al. revealed the high concentration of granulysin in TEN blister fluid. These mediators cause extensive epithelial keratinocyte apoptosis and necrosis (Stern and Divito, 2017). According to recent research, granulysin correlates with illness severity and can be used as a diagnostic test and therapeutic target. Serum IL-15 has been found as an SJS/TEN biomarker (Clayberger and Krensky, 2003; Castillo and Schluns, 2012). Cytotoxic lymphocytes (CTLs) and natural killer cells (NK cells) use IL-5 to differentiate and dominate effector function (Hashizume et al., 2016). The IL-5 signal is sent to the JAK-STAT pathway, which is then followed by the PI3K/AKT/mTOR pathways. In contrast, granulysin expression in CTLs and NK cells is dependent on IL-15 (Yuliwulandari et al., 2017) (Tapia et al., 2004). SJS/TEN patients had IL-17 expression cells in the peripheral blood and fluid blisters, as well as CCR6+ Th17 lymphocyte infiltration in the skin (Bocquet et al., 1996). Human keratinocytes may produce more pro-inflammatory cytokines when IL-17 and IFN- are coupled (Bocquet et al., 1996). CCL27 (CTACK), a keratinocyte-specific chemokine, attracts CCR 10 + lymphocytes to the epidermis. CCR 10 + lymphocytes can be found in high numbers in the peripheral blood during the acute phase of SJS/TEN (Husain et al., 2013).

According to current data, DRESS is caused by a combination of genetic predisposing factors, mainly HLA alleles, viral reactions (HHV-6, HHV-7, EBV, CMV) (Descamps et al., 2001; Ogawa et al., 2013; Cho et al., 2017), and immune system changes, including changes in the drug detoxifying enzyme pathway. These viral reactivations contribute to illnesses being more severe and lasting longer (Ogawa et al., 2014; Tsai et al., 2019). CD4^+^, CD8^+^, Tregs, and monomyeloid cells make up DRESS drug-specific T cells (plasmacytoid dendritic cells, monocyte). CCL17/TARC is a chemokine produced by dermal dendritic cells (DC), attracting CCR4+ Th2 lymphocytes to the skin (Yang et al., 2017). Th2 T cells and innate type 2 lymphocytes (ILC2) produce large quantities of IL-5, which are eosinophil-specific differentiation factors and contribute to the development of eosinophilia (Yang et al., 2017). CD4^+^ T cells that produce IL-4 and IL-13 are also involved. Th2 cell frequencies in DRESS patients are closely related to blood TARC levels, which are linked to HHV reactivation (Yang et al., 2017). ILC2 reflects a high serum concentration of soluble ST2 and IL-33 in the blood and skin of DRESS patients (Carr et al., 2016) *via* expressing the IL-33 receptor ST2 pathway. In the acute stage of DRESS, IL-33 has also been considered a possible biomarker of severity (Carr et al., 2016). DAMP, also known as an alarmin, is an endogenous agonist of pattern-recognition receptors (PRRs) produced by wounded tissue to activate a different immune system (Shear and Spielberg, 1988).

The DAMP/alarmin family includes HMGB1, a non-histone chromatin protein. Transcriptional regulation and extracellular stimulation of inflammation, including recruitment and activation of immunocompetent cells, are two of these dual functions. In the active phase of DRESS, HMGB1 was identified in blood and skin lesions, as well as in the serum and blister fluid of SJS/TEN (Schaerli et al., 2004). Changes in immunological response and metabolic enzymes in the liver, such as the cytochrome P450 (CYP450) enzyme, result in an accumulation of toxin metabolites, causing necrosis and apoptosis (Britschgi and Pichler, 2002).

IL-8/CXCL8 and granulocyte-macrophage colony-stimulating factor (GM-CSF) are produced by drug-specific CD4^+^ T cells for the AGEP (Kabashima et al., 2011). IFN- and TNF-producing T cell clones released IFN and TNF (Kabashima et al., 2011), and a small proportion secreted IL-4 and IL-5. In the skin, IL-8/CXCL8, a neutrophil chemotactic factor, may cause neutrophil and T-cell recruitment to the epidermis, resulting in the accumulation of numerous sterile pustules, as well as recruiting, activating neutrophil from the bone marrow to the peripheral blood, resulting in leukocyte exocytosis and neutrophilia (Romagnani et al., 2009). These recruited neutrophils may be protected from apoptosis by GM-CSF (Kabashima et al., 2011). The cytokines IL-17 and IL-22 are released during the Th17 immune response. Th17 cells have been observed in the skin of AGEP patients, as well as increased serum IL-22 and circulating Th17 cells in the serum (Hadavand et al., 2020). IFN and TNF, which drive surrounding keratinocytes to create IL-8, are connected to IL-17. Furthermore, Th17 cells are known for expressing the C-C motif chemokine receptor- (CCR-) 6. CCR6+ lymphocytes also invaded the afflicted skin (Meier-Schiesser et al., 2019a). A genetic mutation of AGEP pathogenesis involves the IL-36, IL-36 Ra axis. T cells and macrophages produce IL-36 in the skin, whereas monocytes produce IL-36 in the peripheral blood mononuclear cells (PBMCs). This alteration could increase express of various pro-inflammatory cytokines and chemokines such as IL-1, IL-6, and CXCL8/IL-8 production (Meier-Schiesser et al., 2019b; Hadavand et al., 2020).

### HLA and SCARs

The “Hapten concept” or “Haptenization” was first introduced by Landsteiner and Jacobs (1936). As per this model, the hapten is a substance that has <1000 Dalton molecular weight capable of triggering the immune response when it binds with larger proteins or peptides. The electrophilic or reactive chemicals or drugs behave as a hapten and initiate the immune response. According to prohapten theory, the hapten can be generated from metabolites after metabolization. Therefore, the non-reactive drug acquires reactivity by virtue of its metabolites. For instance, sulfamethoxazole is prohapten which is converted into a proactive hydroxylamine metabolite by CYP2C9 at first, then into the unstable and readily protein binding nitroso sulfamethoxazole. This prohapten complex can initiate the immunological response as it is processed by an antigen-presenting cell (APC) and presented to T cell through MHC or HLA (Yun et al., 2016). However, the US FDA label for sulfamethoxazole states that patients with a slow acetylator status are at increased risk of hypersensitivity reactions. The reason could be that acetylation is the major metabolic pathway for sulfamethoxazole, as the acetylation is saturated. Then reactive metabolites are generated by CYP450 2C9 (Ikediobi and Schneider, 2021).

In p-i interactions theory, the drug may bind to TCR itself with high affinity at sites that make contact with pMHC resulting in T-cell activation, even in the presence of different peptides or allogeneic HLA (Zanni et al., 1998; Burkhart et al., 2002; Watkins and Pichler, 2013). For example, lidocaine activates T cells almost instantly in the presence of APCs, as revealed by a rapid and sustained intracellular Ca2+ rise (within 1 min). Thus, the immune response can occur before metabolism (Pichler, 2008).

**FIGURE 6:**
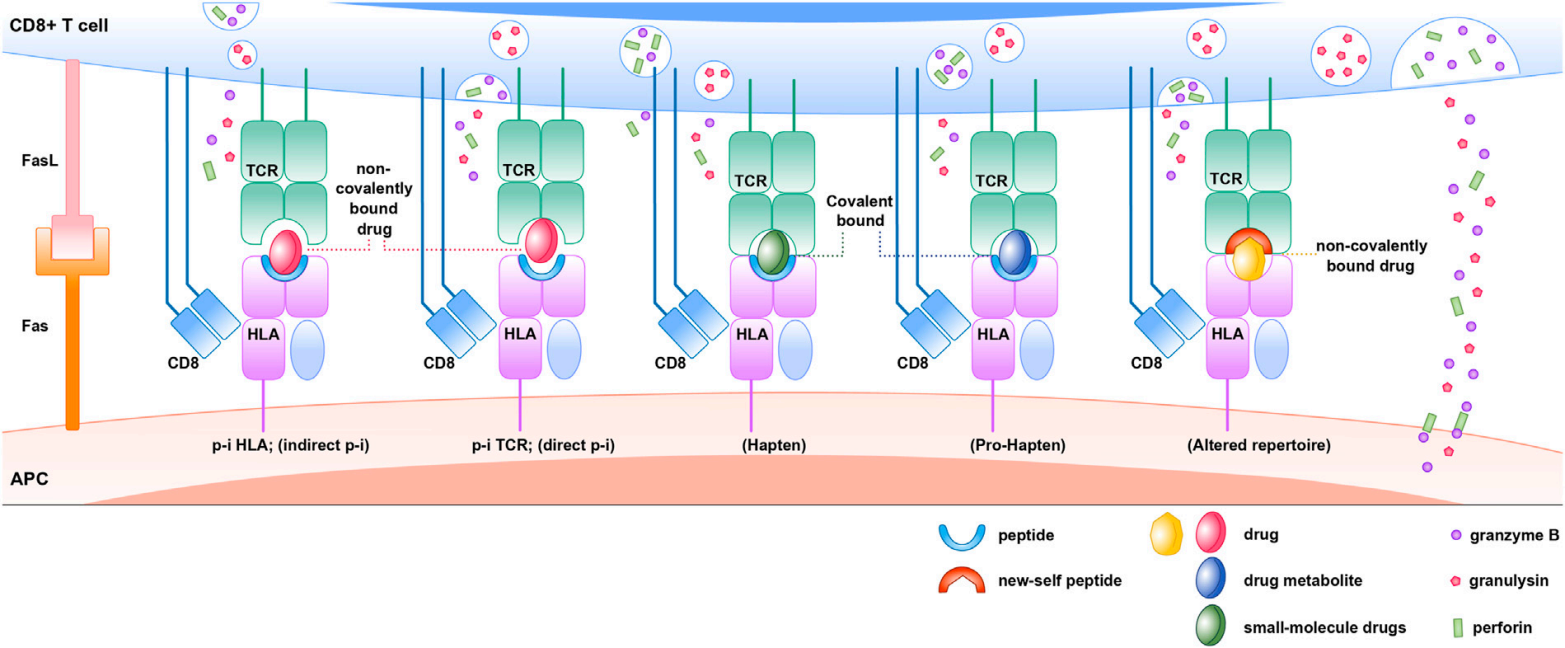
Immune mechanisms of HLA, drug, peptide, and TCR associated drug hypersensitivity reactions.

Repertoire alteration theory proposes that drugs may bind with MHC binding site by non-covalent bond and alter the chemistry of binding cleft and endogenous peptide repertoire. For example, a study by Illing et al. demonstrated that isolated peptides eluted from carbamazepine-treated APCs expressing HLA-B*15:02 were distinct from those obtained in untreated cells. These findings support the altered peptide repertoire model as a feasible explanation for carbamazepine-induced SJS/TEN (Illing et al., 2012). The findings of Norcross et al. (2012) show that abacavir binds non-covalently to HLA-B*57:01 molecule, causing changes in the peptides binding ability of HLA-B*57:01 molecule causing an alteration in the repertoire of endogenous peptides presented to TCR (Figure 6).

However, some concerns about these T cell-mediated SCARs remain unsolved. Those are like why the differences in clinical features and the clinical onset of each SCARs have been observed, the reason for drug-specific re-call reactions years after drug exposure and withdrawal, and the cause of high negative and low positive predictive values. Hence, understanding T cell plasticity and heterologous immunity are critical. The T cell repertoire interacts with several types of antigen to initiate the robust immunological response. Because the human immune system has evolved to have a larger degree of antigenic diversity, this poly-specific TCR identifies several peptides.

Heterologous immunity refers to the ability of a single TCR to recognize various pathogens and protect against the entire microbial universe. Tissue rejection occurs in organ transplantation when the cross-reactive TCR identifies the antigen presented in non-MHC molecules (Gamadia et al., 2004). For example, TCR LC13 can recognize both the HLA-B* 08:01 (EBV epitope) and the endogenous peptide presented in HLA-B* 44:02 and HLA-B* 44:05. The cross-reactivity in the molecular mimicry paradigm is owing to the contact residue’s structure and chemical characteristics being preserved. The structural flexibility of the TCR itself is another mechanism of T cell cross-reactivity. According to this theory, T cells can modify the peptide-MHC interaction by shifting the orientation of CDRs (1–3) *via* an induced-fit mechanism (immune surveillance by CD8αα+ skin-resident T cells in human herpesvirus infection (Zhu et al., 2013).

**FIGURE 7:**
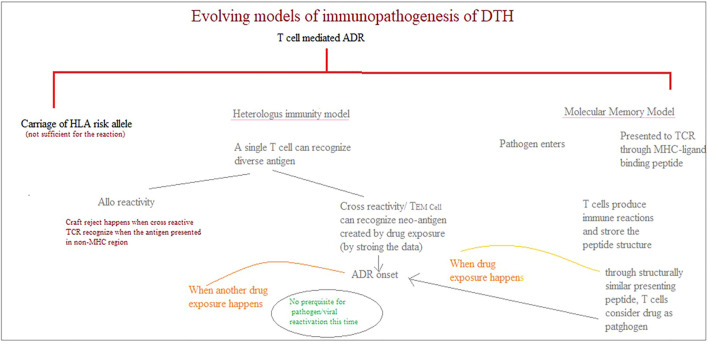
Evolving models of immunopathogenesis of DTH.

Heterologous immunity can also be seen in T cell-mediated hypersensitivity events. According to this paradigm, pathogen-specific effector memory T cells recognize the neo-antigen generated by drug exposure in cross-reactive T cell responses. As a result, viral reactivation or the presence of the pathogen at the initiation of ADR is not required. There is no pathogen reactivation after the initial ADR because the peptide and drug complex activate the T-cell response. This has been verified that HHV replication is not found by viral DNA PCR at an earlier stage of DRESS but is observed after 2 weeks. This reactivation could be because of immune regulation in general. This explains why, while viral reactivation is not the cause of DRESS, it can exacerbate several symptoms of the disease, such as hepatitis and rash, in the absence of a drug (Zhu et al., 2013) (Figure 7).

### Cutaneous Adverse Drug Reaction in Special Populations

#### HIV

CADRs are a prevalent HIV-related complication that may affect the patients’ care plan. There are several reasons behind this. For example, the ART may be the instigators. Aside from that, different factors could be implicated in the onset of CADRs, such as polypharmacy, slow acetylator status, relative glutathione status, CD+4 T-cell counts, latent cytomegalovirus and Epstein–Barr virus infections, and high CD8^+^ T cells count of >460 cells/mm^3^ (Hoosen et al., 2019). The occurrence of cutaneous adverse drug reactions to trimethoprim/sulfamethoxazole (TMP/SMX) is thought to be due to a glutathione deficiency (van der Ven et al., 1991). CADRs are common in HIV patients because opportunistic infections, as well as the use of ART, anti-TB, and NSAIDs, are common. According to a retrospective study conducted in a dermatology ward, 21.8% of CADR cases are anti-TB related, with 92 percent infected with HIV.

Abacavir is a nucleoside nucleotide reverse transcriptase inhibitor (NRTI) available as monotherapy or in fixed-dose combination with lamivudine and lamivudine/zidovudine. Hypersensitivity reaction is the most common skin reaction associated with abacavir. Tipranavir (protease inhibitor), darunavir (protease inhibitor), etravirine (NNRTI)/raltegravir (integrase inhibitor), and maraviroc (chemokine receptor antagonist) are among the ARTs that can produce CADRs (Borras-Blasco et al., 2008). An abacavir hypersensitivity reaction is significantly connected to the presence of the HLA-B*57:01 variant. Likewise, HLA-B* 13:01 is found to be associated with co-trimoxazole-induced CADRs in Thai, Malays, and Taiwanese populations (Kiertiburanakul et al., 2015). As a result, early detection of the causative drug and its correlation with susceptible genetic and non-genetic predictors can aid in the prevention of reactions from progressing.

### Autoimmune/Immune Disorders/Systemic Lupus Erythematous

Drug-induced cutaneous reactions are quite common among patients with autoimmune disorders or patients with immune dysregulations. A retrospective study found that drug allergies are more common among patients with SLE than controls (*p* < 0.01) (Becker, 1973). Another study investigating the imputable drugs of CADRs found 48 patients who suffered from human immunodeficiency virus (HIV) infection (19%), connective tissue disease (10%), and viral or autoimmune hepatitis (12%) (Fiszenson-Albala et al., 2003).

A 10-month prospective cohort study found that immuno-suppressed patients were most frequently affected. Patients with systemic lupus erythematosus (SLE) had a 4.68 greater chance of having a CADRs (CI 95% = 1.794–12.186, *p* = 0.001). Patients with AIDS had a risk of 8.68% (CI 95% = 2.18–33.19, *p* = 0.001). Patients with non-Hodgkin lymphoma had a higher probability of having CADRs (Hernández-Salazar et al., 2006). A tertiary center in India evaluated and their correlation with autoimmune disorders and found a strong relationship between CADRs and autoimmune diseases (*p*-value = 0.004) (Rana et al., 2021). Immune dysregulations present in autoimmune disorders are the major reason for this kind of delayed-type T-cell-mediated hypersensitivity reaction. On the contrary, some studies suggest that autoimmune disorders are the long-term complications of CADRs (Kano* and Shiohara, 2013).

### Tumor Lysis Syndrome

TLS is a life-threatening hemato-oncological emergency (Howard et al., 2011) Hyperuricemia, hyperkalemia, hyperphosphatemia, hypocalcaemia, and metabolic acidosis can all result from the fast disintegration of cancer cells. Systemic tumor lysis syndrome and spontaneous (idiopathic) cutaneous tumor lysis syndrome are the two most common types (Cohen et al., 2021). This cutaneous TLS might be idiopathic (unrelated to therapy) or therapy-related. The clinical signs of cutaneous tumor lysis syndrome are ulcers or necrosis inside the tumor mass.

Rapid tumor cell death is the main diagnostic criteria of TLS. According to an earlier study, bevacizumab, as part of a combined anti-neoplastic treatment, was connected to cutaneous tumor lysis syndrome in people with metastatic breast cancer (Cottu et al., 2011; Ono et al., 2013; Sharma and Marcus, 2013; Kijima et al., 2015). By blocking the vascular endothelial growth factor, bevacizumab damages the tumor vascular system that leads to the breakdown of metastatic tumor cells in the dermis.

Cutaneous TLS due to anti-neoplastic therapies has been reported in T lymphoma patients, especially for bexarotene, vorinostat, and fenofibrate, or retinoids. Methotrexate has also been associated with cutaneous TLS (Hsu et al., 2006; Steinhoff et al., 2008; Pfistershammer et al., 2010; Sánchez et al., 2019).

### CADRs in Pediatric Patients

Differences in the drug metabolism and lack of substantial evidence on drug efficacy and toxic effects could be the reason for the occurrence of CADRs in the pediatric population. Most of the time, drug-induced cutaneous adverse reactions are mistakenly diagnosed as viral exanthema. A retrospective study conducted in India reported 33 CADRs among children. Most of the reactions were MPE, but one death was reported in AED-induced TEN cases. 14% of these reactions were due to antimicrobial, 5% were due to AEDs. SJS was reported mainly with AEDs and antimicrobial (Satyanarayana et al., 2020). Another study from Turkey, whose research population was with 1.99 years of mean age group, found that the most common form of CADRs was urticarial angioedema, which was produced mostly by antibiotics, analgesics, anti-inflammatory, and antipyretic medicines (AAA) (Dilek1 et al., 2014).

## Genetic Markers Associated with Cutaneous Adverse Drug Reactions to the Clinical PG_x_ Implementation in Thailand

Following the accomplishment of the human genome project in 2001, the fields of pharmacogenomics and personalized medicine have exploded globally. In 2004, the Thailand Centre of Excellence for Life Sciences began working on several initiatives. In Thailand, pharmacogenetic biomarkers linked to the top 10 ADRs-involved medicines, such as AEDs, antiretroviral therapy (ART), and allopurinol, have been widely reported. In the Thai population, HLA-B* 15:02 and HLA-B* 58:01 were linked to CBZ-induced SJS/TEN and allopurinol-induced SCARs, respectively (Tassaneeyakul et al., 2009b; Tassaneeyakul et al., 2010b). Patients found to be negative for HLA-B* 15:02 but still developed SCARs were associated with HLA-B* 15:13 and HLA-B* 15:21 in the same serotype *75* (Jaruthamsophon et al., 2017; Sukasem et al., 2021). Other than these drugs, other AEDs such as lamotrigine (HLA-A* 02:07, HLA-A*33: 03, HLA-B*15:02, HLA-B*44:03) (Napatrupron et al., 2017), phenytoin (HLA-B*15:02, HLA-B*15:13, HLA-B*56:02/04) (Kano et al., 2010), oxcarbamazepine (HLA-B* 15:02) (Chen et al., 2017), and phenobarbital (HLA-B* 13:01 and HLA-B* 51:01) (Manuyakorn et al., 2016b) were studied well for their association with HLA alleles and cutaneous adverse reactions. Other than HLA alleles, *CYP2C9*, *CYP2C19*, and omeprazole were other predictors of PHT-induced CADRs. Likewise, *CYP2C19* was associated with phenobarbital-induced CADRs (Manuyakorn et al., 2016b).

Apart from AEDs, cotrimoxazole- (CTX-) induced DRESS has been connected to HLA-B* 13:01 in the Thai population, while CTX-induced SJS/TEN has been linked to HLA-B* 15:02 in the same group (Sukasem et al., 2020b). HLA-B*13:01 has been linked to CADRs caused by dapsone, indicating phenotypic non-specific reactivity (Tangamornsuksan and Lohitnavy, 2018). In the Thai population, HLA-DQB1*03:01 has been associated with DPP4 inhibitor-induced bullous pemphigoid (Chanprapaph et al., 2021).

The risks can be minimized by genotyping the susceptible HLA alleles in the vulnerable population. HLA-A* 31:01, HLA-B* 15:02, HLA-B* 57:01, and HLA-B* 58:01 alleles were evaluated in Thailand even before administering such medications to avoid SCARs. In Thailand, the cost-effectiveness of pharmacogenetic testing was evaluated for two distinct alleles: HLA-B* 15:02 and HLA-B* 58:01. Thailand, China, and Malaysia are among the middle-income countries that reimburse the expense of genetic testing. Only Singapore and Thailand have all of the necessary elements for PGx implementation ready and available.

## Severe Cutaneous Adverse Drug Reactions in Thai Population: Lessons Learned from ThaiSCAR

ThaiSCAR is a multicenter registry of patients with severe cutaneous adverse reactions (SCARs) from drugs among tertiary medical institutes/hospitals in Thailand. ThaiSCAR is aims to study etiologies, clinical characteristics, therapeutic outcomes, quality of life, and the values of laboratory techniques for culprit drugs identification in Thai patients suffering from SCARs. The network was established in October 2013 with eight participating medical schools and hospitals from all country regions. The registry includes patients aged more than 18 years who suffered from SCARs, SJS/TEN, DRESS, AGEP, and GBFDE. The diagnosis is made based on the RegiSCAR diagnostic criteria (Bastuji-Garin et al., 1993; Sidoroff et al., 2001; Kardaun et al., 2007; Lipowicz et al., 2013). Only cases considered probable or definite are included. Importantly, all cases are assessed by dermatologists/allergists at their point of care.

A multidisciplinary team including immunologists, pharmacogeneticists, allergists, and ophthalmologists, in addition to dermatologists, were incorporated into the data gathering of the registry. The correct culprit drug identification is of paramount importance in the management of SCARs. The methods used to identify the causative drugs in the registry are lymphocyte transformation test (LTT) and enzyme-linked immunospot assay (ELISpot) to detect drug-specific IFN-γ-releasing cells from the patients’ peripheral blood mononuclear cells (PBMC). We demonstrated that IFN-γ ELISpot assay yielded positive results in 73.9% of SCARs patients while that of LTT was 52.2%. Interestingly, in this study, the ELISpot assay was undertaken within 2 weeks after the incidence of SCARs, while LTT was performed between 3 and 6 months after the skin eruptions had improved. Therefore, during the acute phase of the disease, ELISpot assay may be a beneficial tool (Suthumchai et al., 2018b). Our further study has confirmed the utility of IFN-γ ELISpot in SCARs. The culprit drugs can be identified in 45.7% (53 of 116) of the patients by the technique. The positive rate was highest in DRESS (50.0%), followed by SJS/TEN (46.0%) and AGEP (31.3%), respectively (Klaewsongkram et al., 2019).

Ocular involvement in SJS/TEN is one of the most devastating manifestations of the disease. Clinical features and serum biomarkers to predict acute severe ocular complications (SOCs) would be crucial to aid in the management of clinicians. From our ThaiSCAR recent study, body surface area detachment of ≥10% was a significant predictive factor for acute SOCs. In addition, serum levels of inflammatory mediators, especially S100A8/A9 and granulysin, showed a significantly higher trend in the severe group compared to those of non-severe individuals (Panpruk et al., 2021).

A study conducted using data obtained from the ThaiSCAR registry shows a slight female preponderance (female: male, n = 174:147, 54.2:45.8%). Of 155 patients with SJS/TEN, anticonvulsants, allopurinol, beta-lactams, sulfonamides/sulfones, and anti-tuberculosis drugs were the leading culprits. One hundred and fifteen (115) patients were seen with DRESS, and anticonvulsants, allopurinol, sulfonamides/sulfones, anti-tuberculosis drugs, and antiretroviral drugs were the most common causative agents. AGEP was observed in 46 patients, with beta-lactam and non-beta-lactam antibiotics being the most common. Lastly, GBFDE was seen in five patients from beta-lactams, sulfonamides/sulfones, non-beta-lactam antibiotics, and analgesic drugs (Sukasem et al., 2020c). The study also found that skin lightening cream was the reason for one case of overlap SJS-TEN. The oral supplements available for lighter skin in Thailand usually contain glutathione, tomato extract, and vitamin C (Chottawornsak et al., 2021).

## Future Perspective

Consensual diagnostic criteria are also necessary for severe cutaneous adverse effects other than SJS/TEN and DRESS. Likewise, regular updates on diagnosis and the relevance of clinical risk factors are required for the accepted diagnostic criteria for SJS/TEN and DRESS. For example, the established diagnostic criteria contained unambiguous clinical manifestations for skin and mucous membrane involvement, showing the involvement of internal organs, particularly liver involvement, is still difficult. The pattern of liver injury must be thoroughly investigated and updated in the criteria that can distinguish SCARs with (drug-induced liver injury) DILI from DILI without SCARs (Sanabria-Cabrera et al., 2019). Similarly, aside from the causality assessment tools, there are no specific or collective tools available to evaluate SCARs in relation to liver injury. Because of this, less effective treatment for SCARs with systemic involvement may be chosen (Villanueva-Paz et al., 2021).

Although many pharmacogenetic biomarkers are being studied in several populations, how these data can be used at the maximum level to predict and prevent SCARs in the real clinical world is the issue for future research to explore. Likewise, more research is needed to investigate various barriers to PGx implementation such as bringing awareness about PGx and reeducating the PGx to sectors of the health care system. Attempts to incorporate PGx into government healthcare plans and insurance schemes could be very useful to the literature. Similarly, the pharmacoeconomic aspects of PGx, such as the cost and clinical advantages of single genetic testing and the range of panel testing, must be thoroughly investigated. This evidence will aid patients in obtaining funding for genetic testing through insurance and government healthcare programs.
